# Salt Appetite and Aging

**DOI:** 10.5935/abc.20190186

**Published:** 2019-09

**Authors:** Celso Amodeo

**Affiliations:** Setor de Cardiopatia Hipertensiva da Disciplina de Cardiologia da UNIFESP, São Paulo, SP - Brazil

**Keywords:** Hypertension, Salt Tolerance, Food Preferences, Sodium Chloride, Dietary/adverse effects, Aged, Aging

The Villela et al.^1^ study showed greater preference and salt intake by hypertensive individuals over the normotensives one regardless of age.

A relationship between higher salt preference and male gender and alcohol consumption was observed. Most sensitivity to salt is the elderly and afro-descendant hypertensive people.

Salt sensitivity increase with advancing age.^2^ One of the reason is that the kidney is less able to either conserve sodium in response to dietary restriction or remove sodium after excess intake.^3,4^ Both aging rats and humans have a blunted ability to excrete an acutely administered sodium load.^3,5,6^

It has long been recognized that reducing dietary salt content has better blood pressure control. Also, it is now known that there are different degrees of salt sensitivity in the hypertensive and normotensive population. Therefore, in non-pharmacological treatment of hypertension, salt reduction is one of the most important interventions.^4^ However, diets restricted in salt are not well tolerated by most patients. Many attempts to substitute salt for other substances have been employed. The addition of oregano to the foods in the Villella’s study resulted in the preference for the lower salt samples in all groups studied.

Just as hypertension is a multifactorial disease, the phenomenon of salt sensitivity is also multifactorial involving genetic, environmental and aging-related aspects. Therefore, salt sensitivity also increases with age and is more marked in African Americans, obese, and patients with metabolic syndrome and/or chronic kidney disease.^6^ Thus, excess salt intake over many years may probably play a greater role in the development of hypertension in these groups. Salt-sensitive normotensives may be more likely to develop hypertension.

The mechanisms of salt sensitivity are not yet fully understood. The lower activation of the renin-aldosterone mechanism may explain the greater fall in BP with reduced sodium intake among the elderly, African Americans, and patients with CKD. Impairment of renal sodium excretion may initially lead to volume expansion and then hypertension.

Multiple genes have been implicated in the pathogenesis of hypertension, including those that regulate sodium absorption, which should undoubtedly participate in the phenomenon of salt sensitivity.

Therefore, the conclusions of the study by Villela et al.^1^ bring new information about the salt preferences of different extracts of our population as well as the possibility of decreasing the salt content in foods with substitution by other spices such as oregano.
